# Prevalence and Antifungal Susceptibility of Pathogenic Yeasts in China: A 10-Year Retrospective Study in a Teaching Hospital

**DOI:** 10.3389/fmicb.2020.01401

**Published:** 2020-07-03

**Authors:** Yinggai Song, Xianlian Chen, Yan Yan, Zhe Wan, Wei Liu, Ruoyu Li

**Affiliations:** ^1^Department of Dermatology and Venereology, Peking University First Hospital, Beijing, China; ^2^Research Center for Medical Mycology, Peking University, Beijing, China; ^3^National Clinical Research Center for Skin and Immune Diseases, Beijing, China; ^4^Department of Laboratory Medicine, The First Affiliated Hospital of Zunyi Medical University, Zunyi, China; ^5^Department of Laboratory Medicine, Peking University First Hospital, Beijing, China

**Keywords:** yeast isolate, *Candida* spp., invasive fungal infection, candidemia, antifungal susceptibility, Sensititre YeastOne, triazoles, echinocandins

## Abstract

To determine the dynamic changes of pathogenic yeast prevalence and antifungal susceptibility patterns in tertiary hospitals in China, we analyzed 527 yeast isolates preserved in the Research Center for Medical Mycology at Peking University, Beijing, China, between Jan 2010 and Dec 2019 and correctly identified 19 yeast species by matrix-assisted laser desorption ionization time-of-flight mass spectrometry (MALDI-TOF-MS) and ribosomal DNA sequencing. Antifungal susceptibility testing was performed following a Sensititre YeastOne colorimetric microdilution panel with nine clinically available antifungals. The Clinical and Laboratory Standards Institute (CLSI)-approved standard M27-A3 (S4) and newly revised clinical breakpoints or species-specific and method-specific epidemiological cutoff values were used for the interpretation of susceptibility test data. In this study, although *Candida albicans* was the predominant single species, non-*C. albicans* species constituted >50% of isolates in 6 out of 10 years, and more rare species were present in the recent 5 years. The non-*C. albicans* species identified most frequently were *Candida parapsilosis sensu stricto*, *Candida tropicalis*, and *Candida glabrata*. The prevalence of fluconazole and voriconazole resistance in the *C. parapsilosis sensu stricto* population was <3%, but *C. tropicalis* exhibited decreased susceptibility to fluconazole (42, 57.5%) and voriconazole (31, 42.5%), and 22 (30.1%) *C. tropicalis* isolates exhibited wild-type minimum inhibitory concentrations (MICs) to posaconazole. Furthermore, fluconazole and voriconazole cross-resistance prevalence in *C. tropicalis* was 19 (26.1%). The overall prevalence of fluconazole resistance in the *C. glabrata* population was 14 (26.9%), and prevalence of isolates exhibiting voriconazole non-wild-type MICs was 33 (63.5%). High-level echinocandin resistance was mainly observed in *C. glabrata*, and the prevalence rates of isolate resistance to anidulafungin, micafungin, and caspofungin were 5 (9.6%), 5 (9.6%), and 4 (7.7%), respectively. Moreover, one *C. glabrata* isolate showed multidrug resistant to azoles, echinocandins, and flucytosine. Overall, the 10-year surveillance study showed the increasing prevalence of non-*C. albicans* species over time; the emergence of azole resistance in *C. tropicalis* and multidrug resistance in *C. glabrata* over the years reinforced the need for epidemiological surveillance and monitoring.

## Introduction

A growing population of immunocompromised patients has resulted in frequent diagnoses of invasive fungal infections (IFIs), including those caused by unusual yeasts (Dabas et al., 2017). Invasive candidiasis is a growing concern worldwide with high morbidity and mortality; it affects patients of all ages, including patients with malignancies, HIV-negative immunocompromised (IC) patients, and non-immunocompromised (NIC) patients among those who are critically ill, often admitted to an intensive care unit (ICU), or diagnosed with diabetes mellitus or uncontrolled hyperglycemia requiring invasive mechanical ventilation (Berdal et al., 2014; Bitar et al., 2014; Epelbaum and Chasan, 2017; Alves et al., 2018; Ding et al., 2018; Mantadakis et al., 2018). Although *Candida albicans* is still the leading cause of fungemia, epidemiological switch and species distribution have shifted toward non-*C. albicans* in recent decades, especially in patients with severe health conditions (Canton et al., 2012; Pfaller et al., 2015; Taei et al., 2019; Xiao et al., 2020). There are considerable geographic and population variability in the prevalence of pathogenic *Candida* species; for example, *Candida tropicalis* is frequently isolated in Asia and South America, and *Candida glabrata* has a high frequency in North and Central Europe and the United States, specially among elderly people (Castanheira et al., 2016; Pappas et al., 2018). *C. glabrata* is also generally more common among individuals >60 years of age and among recipients of solid organ transplant (Cleveland et al., 2015; McCarty and Pappas, 2016). The growing number of non-*C. albicans* species might be connected to former exposure to polyenes and azoles, use of indwelling catheters, malignancies, age, the improved biochemical and molecular diagnostic methods in laboratories, and geographical regions (Diekema et al., 2012; Pfaller et al., 2012; Kullberg and Arendrup, 2015; McCarty and Pappas, 2016; Perlin et al., 2017; Pappas et al., 2018).

The incidence of non-*C. albicans* species infection is increasing compared with that of *C. albicans*, and antifungal resistance was generally uncommon; however, azole resistance occurs in several species, such as *C. glabrata*, *C. tropicalis*, and *Candida krusei* (Berdal et al., 2014; Chapman et al., 2017). There have been reports of outbreaks of azole-resistant *Candida parapsilosis sensu stricto* in adult ICUs in Brazil and in African neonatal ICUs (NICUs) (Neji et al., 2017; Thomaz et al., 2018). In addition, susceptibility to antifungals is variable among species of the *C. parapsilosis* complex (Brilhante et al., 2018). Moreover, in 2009, a new fluconazole-resistant species, *Candida auris*, was identified in East Asia and has now been isolated on five continents, showing high morbidity and mortality (Spivak and Hanson, 2018). Echinocandins have been used as preferred antifungals for most candidemia and invasive candidiasis (Mora-Duarte et al., 2002; Andes et al., 2012; Pappas et al., 2016), but echinocandin resistance among *Candida* species is rising. *C. glabrata* shows cross-resistance to azoles, limiting therapeutic options. The recent emergence of multidrug-resistant *Candida* species further complicates the selection of antifungal therapy owing to the lack of data that can guide therapy (Pfaller et al., 2011a, b; Alexander et al., 2013; Lewis et al., 2013; Castanheira et al., 2014). These findings highlight the importance of epidemiological and laboratory studies of invasive candidiasis in hospitals (Canela et al., 2018).

Rapid species identification and antifungal susceptibility tests are essential for the treatment of IFIs. Matrix-assisted laser desorption ionization time-of-flight mass spectrometry (MALDI-TOF-MS) combined with nucleotide sequencing is a reliable approach for identifying yeast isolates (Maldonado et al., 2018; Kal Çakmaklıoğulları et al., 2019). The commercial Sensititre YeastOne (SYO) test is widely used to determine the susceptibility of *Candida* spp., and it provides an easy and affordable alternative to the Clinical and Laboratory Standards Institute (CLSI) or the European Committee on Antimicrobial Susceptibility Testing (EUCAST) standard broth microdilution methods (Cuenca-Estrella and Rodriguez-Tudela, 2010; Pfaller, 2012; Posteraro and Sanguinetti, 2014; Posteraro et al., 2015). For *Candida* spp., the results of the SYO test are generally in agreement with those obtained by EUCAST and CLSI reference methods, and some method-dependent epidemiological cutoff values (ECVs) were also defined following the criteria recently published by the CLSI (Eschenauer et al., 2014; Espinel-Ingroff et al., 2015, 2019; Posteraro et al., 2015; Perlin et al., 2017).

We believe that knowledge of invasive yeast infection epidemiology in a tertiary hospital in China, including geographical variability and dynamics of susceptibility to the clinical available antifungal drugs, is essential for guiding antifungal treatment protocol development. The objectives of this study were to determine the species distribution and *in vitro* antifungal susceptibility patterns of yeast species collected successively for 10 years from a tertiary teaching hospital at Beijing, China.

## Materials and Methods

### Study Design

This study was a retrospective, laboratory-based study of invasive yeast infections with its inception in Jan 2010 and end in Dec 2019. The 527 yeast isolates came from 514 patients, with more than one episode of candidiasis in some patients. The isolates were preserved at the Research Center for Medical Mycology at Peking University First Hospital, Beijing, China. It is a “rank-A tertiary” hospital with 64 wards and has a total of 1,835 beds and admits more than 86,000 patients per year and provides care for different types of patients across the country. Surgical patients account for approximately 51.2% of the hospital population.

For each episode of yeast isolation (see the criteria for study inclusion below), the information collected included the patient’s age and gender, the patient’s classification (inpatient or outpatient), and the ward location [e.g., emergency department (ED), surgical, medical, and ICU] of the patient at the time of collection of the sample. The date of sample collection, the specimen type, the body site of isolation, and the initial species identification made by the referring laboratory were also recorded. In this study, all isolates were isolated from normally sterile clinical samples of inpatients.

### Criteria for Study Inclusion

We collected all *Candida*, *Cryptococcus*, and other yeast isolates recovered from blood; other sterile body fluids, including ascitic fluid and peritoneal dialysate fluid, pus, and tissue from patients with invasive yeast infections (De Pauw et al., 2008) were included in this study. Yeast isolates from bronchoalveolar lavage (BAL) fluid samples, central venous catheter (CVC) tips, and the gastrointestinal tracts [e.g., biliary tract fluid (aseptically collected)] of patients with invasive infections were tested; however, yeast isolates from sputum, urine, and the genital tract and others considered colonizers were excluded. Isolates of the same species and of the same susceptible or resistant biotype profile from the same site of a given patient isolated at a different time were considered duplicates and also excluded.

### Species Identification

All yeast isolates were reidentified to the species level by MALDI-TOF-MS using the MALDI Biotyper RTC 4.0 software (Bruker Daltonik) by rastering the target position. For any isolate with no identification or uncertain identification (e.g., low confidence value) results by MALDI-TOF-MS and for all isolates identified within the *C. parapsilosis* complex (i.e., *C. parapsilosis sensu stricto*, *Candida metapsilosis*, and *Candida orthopsilosis*) and *C. glabrata*, sequencing of the internal transcribed spacer (ITS) rDNA (ITS1/ITS4) region was performed for definitive species identification.

### Antifungal Susceptibility Testing

An SYO YO10 panel tray (Thermo Scientific, Cleveland, OH, United States) was used for antifungal susceptibility tests. The plate contains serial twofold dilutions of amphotericin B (0.12–8 mg/L), flucytosine (0.06–64 mg/L), fluconazole (0.12–256 mg/L), itraconazole (0.015–16 mg/L), voriconazole (0.008–8 mg/L), posaconazole (0.008–8 mg/L), anidulafungin (0.015–8 mg/L), micafungin (0.008–8 mg/L), and caspofungin (0.008–8 mg/L).

Antifungal susceptibility testing was performed following the manufacturer’s instructions. *C. parapsilosis* ATCC 22019 and *C. krusei* ATCC 6258 from the American Type Culture Collection were included as control isolates in all experiments. Minimum inhibitory concentrations (MICs) were determined after 24 h of incubation at 35°C for *Candida* spp. and *Trichosporon* spp. and after 72 h of incubation for *Cryptococcus* spp. MIC was defined as the lowest concentration of an antifungal at which the color in the well changed from red (positive, indicating growth) to blue (negative, indicating no growth).

### Interpretation of MIC Results

MIC data were determined using the CLSI-approved standard M27-S4, and interpretation of susceptibility was performed by applying the updated species-specific CLSI clinical breakpoints (CBPs) (CLSI, 2017, Wayne, PA, United States), or epidemiological cutoff values (ECVs) where CBPs were not available (CLSI, 2018, Wayne, PA, United States; Canton et al., 2012; Pfaller and Diekema, 2012; Espinel-Ingroff et al., 2019) were applied (Supplementary Table 1). In the absence of CBPs, isolates were defined as having a wild-type (WT) or a non-WT (NWT) drug susceptibility phenotype according to the ECVs as determined by the CLSI broth microdilution methods and colorimetric SYO method.

### Ethical Approval

This retrospective study was approved by the ethics committee of Peking University First Hospital. The need for informed consent was waived by the Clinical Research Ethics Committee.

### Statistical Analysis

The data were analyzed using SPSS software version 22 for Windows (SPSS, Chicago, IL, United States). Categorical variables were analyzed using the chi-square test or Fisher’s exact test, and continuous variables were analyzed by the Mann–Whitney *U* test. Data were processed using the Kruskal–Wallis test to avoid random significance when comparing several groups. Significance was set as a *P* value of <0.05 (two-tailed). GraphPad Prism version 8.0 (GraphPad, San Diego, CA, United States) was used to perform the analyses.

## Results

### Patient Demographics

A total of 527 yeast isolates were isolated from inpatient wards and preserved at the Research Center for Medical Mycology in a tertiary teaching hospital at Beijing, China. Of the yeast isolates investigated, 201 (38.1%) were isolated from female patients and 326 (61.9%) were from male patients. Patient age ranged from 1 h to 101 years (median, 66.0 years; interquartile range, 48.0–78.0 years). More than half of isolates (400, 75.9%) were isolated from ICU patients, of which 366 (69.4%) were detected in a respiratory ICU, 24 (4.6%) were from a surgery ICU, and the remaining were from a cardiothoracic ICU, a coronary ICU, and a comprehensive ICU. Fifty-eight (11.0%) patients were hospitalized in surgical wards, and four (0.8%) were bone marrow transplant patients. The percentages of the different age groups were as follows: 0–1 year, 39 (7.4%); 2–14 years, 28 (5.3%); 15–49 years, 74 (14.0%); 50–65 years, 119 (22.6%); and over 65 years, 267 (50.7%). There was variation in species with age group. *C. albicans* was most common in patients 0–14 years of age (43, 63.6%), and there were 7 (10.4%)with *C. parapsilosis* complex in this age group. In patients aged over 65 years, *C. albicans* dominated, but the frequency rates of the *C. parapsilosis* complex (53, 19.9%) and *C. glabrata* (30, 11.2%) were slightly higher compared to the overall average (17.3%, 9.9%; *P* > 0.05). Among these, 138 (51.7%) were from ICU patients.

### Species Distribution Over 10 Years

A total of 19 yeast species were identified within the 527 isolates. Our study showed a decrease in the isolation of *C. albicans* and an increasing prevalence of non-*C. albicans* species over time. Although *C. albicans* remained the most frequently recovered species, it comprised less than half of all isolates (259, 49.2%). *C. parapsilosis* complex isolates were 92 (17.5%) (of which 91 were *C. parapsilosis sensu stricto* and 1 was *C. metapsilosis*). *C. tropicalis* (73, 13.9%) was the third most common species, followed by *C. glabrata* (52, 9.9%), *Cryptococcus neoformans* (14, 2.7%), *C. krusei* (12, 2.3%), *Trichosporon asahii* (4, 0.8%), and *Rhodotorula glutinis* (4, 0.8%). The prevalence rates of rare species, such as *Candida lusitaniae*, *Candida guilliermondii*, *Candida inconspicua*, *Pichia anomalus*, *Candida carpophila*, *Candida utilis*, *Candida kefyr*, *Candida lipolytica*, *Cryptococcus curvatus*, and *Trichosporon inkin*, were collectively <1% (Supplementary Table 2).

**FIGURE 1:**
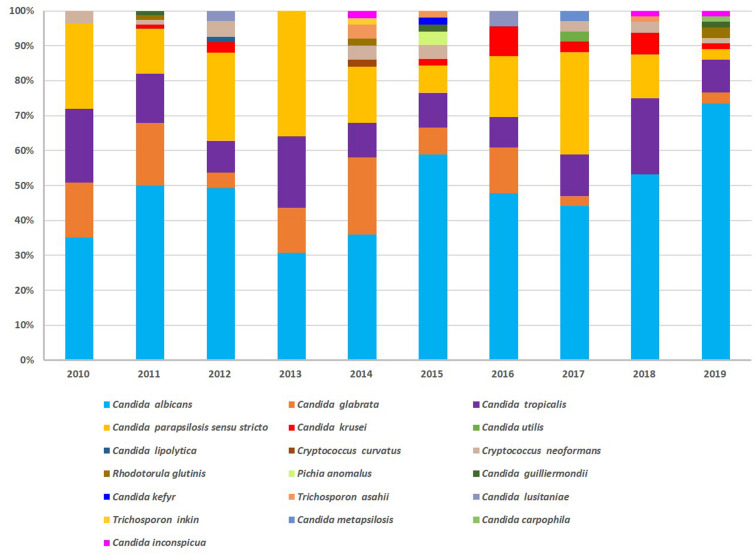
Species distribution over 10 years of surveillance. Yeast species varied across years. More rare species were isolated in the recent 5 years; *C. albicans* was the predominant single species. Non-*C. albicans* species identified most frequently were *C. parapsilosis sensu stricto*, *C. tropicalis*, and *C. glabrata.*

Overall, yeast species isolated varied across years. More rare species were isolated in the recent 5 years; although *C. albicans* was the predominant single species, non-*C. albicans* species constituted >50% of isolates in 6 out of 10 years. The non-*C. albicans* species identified most frequently were *C. parapsilosis sensu stricto*, *C. tropicalis*, and *C. glabrata* (Figure 1).

### Specimen Sources of Yeast Isolates

Concerning sample type, all yeast isolates were isolated from clinical samples, of which blood culture isolates made up 184 (34.9%) and lower respiratory BAL fluid (BALF) accounted for 143 (27.1%). Eighty (15.2%) were detected in ascitic fluid, 39 (7.4%) in pleural fluid, 22 (4.2%) in pus, 19 (3.6%) in cerebrospinal fluid, and 16 (3.0%) in peritoneal dialysis. While CVC tips, bile, tissue, hydrarthrosis, and bone marrow specimens yielded yeast isolates uncommonly (<5%), blood, BALF, and ascitic fluid were the top three most common specimens over 10 years, and yeast species isolated from blood were more frequent than those from other specimens (*χ*^2^ = 527.000, *P* = 0.000) (Supplementary Table 3).

*Candida* species isolates were broadly distributed and made up the majority of the blood culture isolates (174, 94.6%). *Cryptococcus* and non-*Candida* yeast isolates accounted for 5.4% of the yeast isolates from blood cultures. The majority of the yeast isolates from ascitic fluid (78, 97.5%) and pleural fluid (37, 94.9%) were *Candida* spp., and *Candida* spp. were the only yeast isolates from CVC, bile, hydrarthrosis, and tissue. Thirteen (68.4%) of the yeast isolates from CSF samples were *Candida* spp. and exceeded *C. neoformans*. There was a single isolate of *T. asahii* from an ascitic fluid sample (Supplementary Table 3).

Thirteen yeast species were involved in fungemia, of which *C. albicans* was 61 (33.2%). *C. parapsilosis sensu stricto* (48, 26.1%) was the leading species of non-*C. albicans* in candidemia patients, followed by *C. glabrata* (31, 16.8%) and *C. tropicalis* (26, 14.1%), and *C. krusei* (4, 2.2%). The frequency of *R. glutinis*, *C. neoformans*, *T. asahii*, and *P. anomalus* was collectively <1% (Supplementary Table 4).

### *In vitro* Susceptibility to Azoles

The susceptibility of the 527 yeast isolates to antifungals is presented in Table 1. Generally, *C. albicans* and *C. parapsilosis* complex isolates were susceptible or exhibited WT MICs to all four azoles (prevalence of isolates exhibiting resistance or NWT MICs was <5%) during the 10 years. Azole susceptibility was less common among *C. tropicalis* isolates, fluconazole and voriconazole susceptibility was exhibited by only 42 (57.5%) isolates and 31 (42.5%) isolates, respectively; furthermore, 19 (26.1%) isolates were cross-resistant to fluconazole and voriconazole, and 51 (69.9%) *C. tropicalis* isolates exhibited NWT MICs to posaconazole (Supplementary Table 5). The overall prevalence of fluconazole resistance in the *C. glabrata* population was 14 (26.9%), and the prevalence of *C. glabrata* isolates exhibiting NWT to voriconazole was 33 (63.5%). Among *C. krusei*, all isolates showed the WT phenotype to itraconazole, while resistance was more prevalent (8.3%) to voriconazole and posaconazole. However, the remaining non-*C. albicans* species (those with >2.5% prevalence), including *C. guilliermondii*, *C. lusitaniae*, *C. inconspicua*, *C. lipolytica*, *C. utilis*, *C. kefyr*, and *C. carpophila*, exhibited reduced azole susceptibility, and their 50% MIC (MIC50), MIC90, and geometric mean MIC were twofold to 15-fold higher than those of *C. albicans*. The overall cross-resistant prevalence rates in common *Candida* species were 23 (44.2%) in *C. glabrata*, 24 (32.9%) in *C. tropicalis*, 1 (8.3%) in *C. krusei*, and 12 (4.6%) in *C. albicans* (Supplementary Table 5).

**TABLE 1:** Susceptibility of the 527 yeast isolates to nine antifungal agents.

Species	MIC range (mg/L)	MIC_50_^†^ (mg/L)	MIC_90_^†^ (mg/L)	GM (mg/L)	S/WT (%)	Number of isolates (%)				Susceptibility phenotype	
						S	SDD	I	R	WT (%)	Non-WT (%)
***C. albicans* (*n* = 259)**											
Amphotericin B	≤0.12 to 2	0.5	1	0.619	100	–	–	–	–	259 (100)	0
Fluconazole	≤0.12 to >256	0.5	1	1.171	95.0	247 (95.0)	6 (2.3)	–	7 (2.7)	–	–
Voriconazole	≤0.008 to >8	0.008	0.03	0.055	96.1	249 (96.1)	–	6 (2.3)	4 (1.6)	–	–
Posaconazole	≤0.008 to >8	0.03	0.06	0.074	92.3	–	–	–	–	239 (92.3)	20 (7.7%)
Itraconazole	≤0.015 to >16	0.06	0.12	0.114	94.2	–	–	–	–	244 (94.2)	15 (5.8%)
Caspofungin	0.015 to >8	0.06	0.12	0.092	97.3	252 (97.3)	–	5 (1.9)	2 (0.8)	–	–
Micafungin	≤0.008 to >8	0.015	0.015	0.056	98.1	254 (98.1)	–	1 (0.4)	4 (1.6)	–	–
Anidulafungin	≤0.015 to >8	0.06	0.12	0.109	97.7	253 (97.7)	–	2 (0.8)	4 (1.6)	–	–
Flucytosine	≤0.06 to >64	<0.06	0.12	0.224	98.1	–	–	–	–	254 (98.1)	5 (1.9)
***C. parapsilosis* complex (*n* = 92)**											
Amphotericin B	≤0.12 to 2	0.5	1	0.636	100	–	–	–	–	92 (100)	0
Fluconazole	≤0.12 to 64	0.5	1	1.206	95.7	88 (95.7)	2 (2.2)	–	2 (2.2)	–	–
Voriconazole	≤0.008 to 0.5	0.008	0.03	0.055	97.8	90 (97.8)	–	2 (2.2)	0	–	–
Posaconazole	0.008 to 0.5	0.06	0.06	0.076	98.9	–	–	–	–	91 (98.9)	1 (1.1)
Itraconazole	≤0.015 to 0.5	0.06	0.12	0.117	100	–	–	–	–	92 (100)	0
Caspofungin	0.03 to 1	0.5	1	0.094	100	92 (100)	–	0	0	–	–
Micafungin	0.015 to 2	1	1	0.058	100	92 (100)	–	0	0	–	–
Anidulafungin	0.015 to 2	1	2	0.112	100	92 (100)	–	0	0	–	–
Flucytosine	≤0.06 to >64	0.06	0.12	0.221	97.8	–	–	–	–	90 (97.8)	2 (2.2)
***C. tropicalis* (*n* = 73)**											
Amphotericin B	<0.12 to 2	1	1	0.634	100	–	–	–	–	73 (100)	0
Fluconazole	0.25 to >256	2	>256	1.217	57.5	42 (57.5)	5 (6.8)	–	26 (35.6)	–	–
Voriconazole	≤0.008 to >8	0.25	>8	0.055	42.5	31 (42.5)	–	23 (31.5)	19 (26.0)	–	–
Posaconazole	0.03 to >8	0.25	1	0.077	30.1	–	–	–	–	22 (30.1)	51 (69.9)
Itraconazole	0.03 to >16	0.25	1	0.117	78.1	–	–	–	–	57 (78.1)	16 (21.9)
Caspofungin	0.015 to >8	0.06	0.12	0.093	95.9	70 (95.9)	–	1 (1.4)	2 (2.7)	–	–
Micafungin	≤0.008 to >8	0.03	0.03	0.056	98.6	72 (98.6)	–	0	1 (1.4)	–	–
Anidulafungin	0.015 to >8	0.06	0.12	0.111	98.6	72 (98.6)	–	0	1 (1.4)	–	–
Flucytosine	≤0.06 to 0.5	<0.06	0.06	0.224	100.0	–	–	–	–	73 (100)	0
***C. glabrata* (*n* = 52)**											
Amphotericin B	<0.12 to 1	0.5	1	0.64	100	–	–	–	–	52 (100)	0
Fluconazole	0.5 to >256	32	256	1.194	48.1	25 (48.1)	13 (25.0)	–	14 (26.9)	–	–
Voriconazole	0.015 to 8	0.5	2	0.054	36.5	–	–	–	–	19 (36.5)	33 (63.5)
Posaconazole	0.06 to >8	1	>8	0.075	65.4	–	–	–	–	34 (65.4)	18 (34.6)
Itraconazole	0.12 to >16	0.5	2	0.117	92.3	–	–	–	–	48 (92.3)	4 (7.7)
Caspofungin	0.015 to 0.5	0.12	0.25	0.094	71.2	37 (71.2)	–	11 (21.2)	4 (7.7)	–	–
Micafungin	<0.008 to 1	0.015	0.03	0.058	90.4	47 (90.4)	–	0	5 (9.6)	–	–
Anidulafungin	<0.015 to 1	0.03	0.12	0.112	90.4	47 (90.4)	–	0	5 (9.6)	–	–
Flucytosine	≤0.06 to 64	<0.06	<0.06	0.219	98.1	–	–	–	–	51 (98.1)	1 (1.9)
***C. krusei* (*n* = 12)**											
Amphotericin B	<0.12 to 2	1	2	0.616	100	–	–	–	–	12 (100)	0
Fluconazole	32 to 256	64	256	1.202	–	–	–	–	–	–	–
Voriconazole	0.25 to 2	0.5	1	0.056	66.7	8 (66.7)	–	3 (25.0)	1 (8.3)	–	–
Posaconazole	0.25 to 1	0.25	0.5	0.076	91.7	–	–	–	–	11 (91.7)	1 (8.3)
Itraconazole	0.25 to 0.5	0.25	0.5	0.117	100	–	–	–	–	12 (100)	0
Caspofungin	0.06 to 0.5	0.12	0.25	0.094	91.7	11 (91.7)	–	1 (8.3)	0	–	–
Micafungin	0.06 to 0.25	0.12	0.12	0.06	100	12 (100)	–	0	0	–	–
Anidulafungin	<0.015 to 0.12	0.06	0.12	0.114	100	12 (100)	–	0	0	–	–
Flucytosine	2 to 16	8	16	0.229	100	–	–	–	–	12 (100)	0
***C. neoformans* (*n* = 14)**											
Amphotericin B	0.5 to 2	1	2	0.591	–	–	–	–	–	–	–
Fluconazole	0.5 to 4	2	4	1.201	–	–	–	–	–	–	–
Voriconazole	0.015 to 0.06	0.03	0.06	0.059	–	–	–	–	–	–	–
Posaconazole	0.03 to 0.12	0.06	0.12	0.077	–	–	–	–	–	–	–
Itraconazole	<0.015 to 0.12	0.06	0.12	0.116	–	–	–	–	–	–	–
Caspofungin	0.03 to >8	>8	>8	0.095	–	–	–	–	–	–	–
Micafungin	<0.008 to >8	>8	>8	0.059	–	–	–	–	–	–	–
Anidulafungin	0.12 to >8	>8	>8	0.113	–	–	–	–	–	–	–
Flucytosine	0.12 to 32	4	8	0.24	–	–	–	–	–	–	–
**Other species (*n* = 25)^‡^**											
Amphotericin B	0.12 to 2	0.5	1	0.691	–	–	–	–	–	–	–
Fluconazole	0.25 to >256	4	>256	1.295	–	–	–	–	–	–	–
Voriconazole	<0.008 to 8	0.12	1	0.055	–	–	–	–	–	–	–
Posaconazole	0.015 to 2	0.12	1	0.08	–	–	–	–	–	–	–
Itraconazole	0.03 to 1	0.25	0.5	0.124	–	–	–	–	–	–	–
Caspofungin	0.03 to >8	0.5	>8	0.093	–	–	–	–	–	–	–
Micafungin	0.015 to >8	0.5	>8	0.057	–	–	–	–	–	–	–
Anidulafungin	0.015 to >8	1	>8	0.111	–	–	–	–	–	–	–
Flucytosine	<0.06 to 64	0.06	16	0.224	–	–	–	–	–	–	–

*For available clinical break points and/or epidemiological cutoff values, see Supplementary Table 1. ^†^MIC_50_ and MIC_90_: the concentrations at which 50 and 90% of isolates are inhibited. ^‡^Including T. asahii (4), R. glutinis (4), C. guilliermondii (3), C. lusitaniae (3), C. inconspicua (3), P. anomalus (2), C. lipolytica (1), C. curvatus (1), C. utilis (1), C. kefyr (1), T. inkin (1), C. carpophila (1). MIC, minimum inhibitory concentration; S, susceptible; SDD, susceptible-dose dependent; I, intermediate; R, resistant; WT, wild-type.*

The overall prevalence of antifungal agents resistance in 184 blood culture isolates was 25 (13.6%). For *C. tropicalis* and *C. glabrata*, resistance was more prevalent to at least one triazole [19 (73.1%) and 23 (74.2%), respectively]. Triazole cross-resistance prevalence rates were also high in *C. tropicalis* (10, 38.5%) and *C. glabrata* (19, 61.3%) isolates. Resistance to echinocandins was detected in two (3.3%) *C. albicans* isolates and one (3.8%) *C. tropicalis* isolate (Supplementary Table 6).

### *In vitro* Susceptibility to Echinocandins, Amphotericin B, and Flucytosine

Resistance to one or more of the echinocandins was distinctly uncommon among isolates of *C. albicans* (0.8–1.6%), *C. parapsilosis* (0.0%), *C. tropicalis* (1.4–2.7%), and *C. krusei* (0.0%). The prevalence rates of resistance to anidulafungin (5, 9.6%), caspofungin (4, 7.7%), and micafungin (5, 9.6%) were most prominent among *C. glabrata* isolates. No trend toward increasing resistance was seen over time for any of these species, although a higher prevalence of resistant isolates was detected in *C. tropicalis* in 2013–2014 and in *C. glabrata* in 2017–2019. Cross-resistance to two or three echinocandins was observed in three (1.2%) *C. albicans* isolates, five (9.6%) *C. glabrata* isolates, and one (1.4%) *C. tropicalis* isolates.

Amphotericin B also showed good overall activity against common *Candida* species, with WT rates of 100% (Table 1). The MIC50 and MIC90 values of amphotericin B were 1 and 2 μg/ml for *C. krusei* and *C. neoformans*, respectively, and were either 0.5 and 1 μg/ml for *C. lipolytica*, *C. guilliermondii*, *C. carpophila*, and *C. kefyr* species (Table 1). Rare species exhibited amphotericin B MICs of 0.12–2 mg/L (Table 1), which are considered WT for other *Candida* species. Less than 6% of common *Candida* species were of NWT phenotype to flucytosine, while *C. tropicalis* and *C. krusei* were 100% susceptible to flucytosine (Table 1).

### Multidrug Resistance

For species with established ECVs and CBPs, we analyzed antifungal multidrug resistance (i.e., isolates that were resistant or NWT to at least two classes of antifungal agents tested).

Only six isolates (two *C. albicans*, two *C. glabrata*, and two *C. tropicalis*) showed multidrug resistance to two classes of antifungal agents (<3% for each species), of which one *C. albicans* and one *C. glabrata* isolates were multidrug resistant to azoles, echinocandins, and flucytosine.

### Trends in Fluconazole and Voriconazole Resistance Over Time

Trends in fluconazole and voriconazole resistance/NWT for common *Candida* species over time are shown in Table 2. Generally, fluconazole resistance in *C. albicans* slightly increased from 2012 (3.0%) to 2018 (5.9%), but this increase was not statistically significant (*P* > 0.05; Table 2). A significantly higher prevalence of resistant isolates was detected in *C. tropicalis* to fluconazole and voriconazole (frequency of resistance from <10 to 75%) over 10 years, *C. glabrata* to voriconazole (NWT phenotype increased from <25 to 40%; Table 2), and *C. parapsilosis* complex to fluconazole (from <6 to 9%). There were no trends in resistance for other species in this study (Table 2).

**TABLE 2:** Trends of fluconazole and voriconazole resistance or non-wild-type phenotype in *Candida* species over 10 years.

	Resistance (%)									
	2010	2011	2012	2013	2014	2015	2016	2017	2018	2019
**Fluconazole**										
*C. albicans*	0.0	5.1	3.0	0.0	5.6	0.0	0.0	6.7	5.9	0.0
*C. parapsilosis complex*	0.0	0.0	5.9	0.0	0.0	0.0	0.0	9.1	0.0	0.0
*C. tropicalis*	25.0	36.4	16.7	12.5	40.0	60.0	50.0	75.0	35.7	50.0
*C. glabrata*	22.2	35.7	33.3	40.0	18.2	25.0	33.3	0.0	0.0	0.0
*C. krusei*	100.0	100.0	100.0	100.0	100.0	100.0	100.0	100.0	100.0	100.0
**Voriconazole**										
*C. albicans*	0.0	5.1	0.0	0.0	0.0	0.0	0.0	6.7	2.9	0.0
*C. parapsilosis complex*	0.0	0.0	0.0	0.0	0.0	0.0	0.0	0.0	0.0	0.0
*C. tropicalis*	8.3	18.2	0.0	12.5	40.0	40.0	50.0	75.0	35.7	33.3
*C. glabrata*	88.9	71.4	66.7	60.0	36.4	50.0	66.7	0.0	0.0	100.0
*C. krusei*	0.0	0.0	0.0	0.0	0.0	0.0	0.0	0.0	25.0	0.0

## Discussion

The landscape of invasive mycoses is in a continuous evolution with important implications for their diagnosis and treatment. The overall burden remains high, particularly in neonates and the elderly; patients admitted to intensive care units and using prostheses, catheters, or other intravenous devices; those receiving different immunosuppressant treatments or antineoplastic chemotherapy; or transplant recipients (Lortholary et al., 2014; Quindós, 2018; Pfaller et al., 2019). In this 10-year surveillance study, the etiology of invasive candidiasis progressively shifts from *C. albicans* to other species of *Candida*, and the proportion of non-*C. albicans* was over 50% in 6 out of 10 years, of which the *C. parapsilosis* complex, *C. tropicalis*, *C. glabrata*, and *C. krusei* accounted for 43.4% collectively, while *C. albicans* represents more than 50% of isolates in 2018 and more than 70% in 2019 due to the high frequency of *C. albicans* isolated from BALF, with 12 (35.3%) in 2018 and 31 (66.0%) in 2019. The prevalence of *C. parapsilosis sensu stricto* is much higher than *C. tropicalis* and *C. glabrata* (17.3% versus 13.9 and 9.9%). In addition, large nosocomial outbreaks due to *C. parapsilosis sensu stricto* have been reported in NICU patients and adult wards from tertiary hospitals in China (Wang et al., 2016a, b; Qi et al., 2018). Rarer species accounted for 4.5% in this study, including *C. inconspicua*, *C. utilis*, *C. lipolytica*, *C. kefyr*, *C. curvatus*, *C. carpophila*, and *T. inkin*. The species varied gradually over time, and more species were present in the recent 5 years. This increasing prevalence of non-*C. albicans* species over time has been noted in other studies, which is generally considered to be associated with reduced antifungal susceptibilities (Kullberg and Arendrup, 2015; Perlin et al., 2017; Lamoth et al., 2018).

Variation in species distribution was noted among different clinical services and age groups; for example, *C. parapsilosis sensu stricto* was predominant in ICU admissions and among patients aged 65 years and older. This may be because potential risk factors for candidiasis were more common in such patient population, regarding invasive procedures in ICU, the aging of the population, and previous antibiotic exposure (Kullberg and Arendrup, 2015). The trend for increasing *C. parapsilosis* was seen in tertiary university-affiliated hospitals located in southwest China as well (Jia et al., 2018). The picture is somewhat different in a teaching hospital in central China, where the predominant species was *C. tropicalis* (25.1%), followed by the *C. parapsilosis* complex (17.3%) and *C. glabrata* (9%) (Xu et al., 2019). The trend for increasing *C. glabrata* was reported in Australia and in some Latin American and African countries (Nucci et al., 2013; Doi et al., 2016; Govender et al., 2016). The difference showed the geographic and temporal variations in the frequency of *Candida* species.

The epidemiology of candidemia has been described in numerous single-center and population-based surveys conducted worldwide (Guinea, 2014; Yang et al., 2014; Astvad et al., 2018; Lamoth et al., 2018). As our results showed, in the 184 isolates from candidemia patients, the proportion of non-*C. albicans* species exceeded that of *C. albicans* to become the most common among patients infected with candidemia, in accordance with some previous studies from local China, Asia-Pacific regions, and European countries (Liu et al., 2014; Tan et al., 2016; Wang et al., 2016a; Guo et al., 2017; Pappas et al., 2018; Zeng et al., 2019). In addition, the *C. parapsilosis* complex (26.1%) was the second most prevalent *Candida* spp. isolated in our study, the frequencies of which were much higher than those reported in Southern Europe and Southeast Asia (Siri et al., 2017; Teo et al., 2017). Another report analyzed patients with candidemia in the ICU in a teaching hospital in Beijing, with similar trends of the *C. parapsilosis* complex (19.5%), *C. glabrata* (15.9%), and *C. tropicalis* (14.6%) (Xiao et al., 2019), while the prevalence of *C. tropicalis* is much higher in some other reports (Ma et al., 2013; Pfaller et al., 2019; Xiao et al., 2020).

In our study, most *Candida* species isolates were susceptible to the antifungal agents. For example, *C. albicans* and the *C. parapsilosis* complex have low incidences of fluconazole resistance, at 5.0 and 4.3%, respectively. The low fluconazole resistance prevalence among *C. albicans* isolates was consistent with previous reports (Liu et al., 2014; Guo et al., 2017; Brilhante et al., 2018; Zeng et al., 2019). But a trend toward increased resistance or the emergence of naturally resistant species was observed among other *Candida* spp. Our study confirmed that azole resistance was mainly observed in *C. tropicalis* and *C. glabrata* isolates over 10 years. Moreover, the resistance prevalence rates to fluconazole (35.6%) and voriconazole (26.0%) in *C. tropicalis* isolates were much higher than those reported in the single-center study in southwest China (Jia et al., 2018) and CHIF-NET study in China (Guo et al., 2017; Xiao et al., 2020). Notably, 32.9% of *C. tropicalis* isolates were cross-resistant to azoles, but no isolate was multidrug resistant to azoles, flucytosine, and amphotericin B. In *C. glabrata* species, the prevalence rates of fluconazole (26.9%) and voriconazole (63.5%) resistance in this study were much higher than those reported in the United States, Australia, and several European countries, which also have increasing trends in prevalence of *C. glabrata* over time, with 6–15% of isolates being resistant to fluconazole (Chapman et al., 2017; Lamoth et al., 2018; Pfaller et al., 2019). There has been high fluconazole and azole cross-resistance prevalence (>30%) of *C. glabrata* in this study. This 10-year surveillance study identified a sharp increase in fluconazole and voriconazole resistance prevalence of *C. tropicalis* (from <10 to 75%) over 10 years and *C. glabrata* to voriconazole (from <25 to 40%). The *C. parapsilosis* complex showed a slightly increase to fluconazole and voriconazole resistance prevalence (from <6% in 2012 to 9% in 2017), and fluconazole resistance in *C. albicans* increased from 3.0% in 2012 to 5.9% in 2018. *C. glabrata* and *C. tropicalis* are considered to exhibit moderate- to high-level intrinsic azole resistance, with the prevalence and resistance prevalence varying with geographic region (Kullberg and Arendrup, 2015; Fan et al., 2017; Perlin et al., 2017).

Another noteworthy finding of our study was the emergence of echinocandin resistance. High-level echinocandin resistance was mainly observed in *C. glabrata*, with resistance prevalence rates of 9.6% to anidulafungin, 9.6% to micafungin, and 7.7% to caspofungin, all higher than those seen in large-scale surveillance studies in China and Europe (Pham et al., 2014; Klotz et al., 2016; Xiao et al., 2020), and a higher prevalence rate close to or more than 10% in *C. glabrata* is also reported by some institutional studies in North America (Alexander et al., 2013; Farmakiotis et al., 2014). Whether this difference is because of strain types, clinical practice issues, or both is unclear. Notably, one *C. glabrata* isolate was multidrug resistant to azoles, echinocandins, and flucytosine in this study. Previous studies reported that echinocandin resistance in susceptible *Candida* species arises after repeated or long-term exposure (Lortholary et al., 2011; Fekkar et al., 2014; Perlin et al., 2017), and an increase in echinocandin resistance among *C. glabrata* is often accompanied by azole resistance, resulting in multidrug-resistant isolates (Alexander et al., 2013; Pham et al., 2014).

From our study, the increasing prevalence of non-*C. albicans* species over time has been noted. The emergence of azole resistance among non-*C. albicans* is particularly concerning, followed by echinocandin and multidrug resistance among some *Candida* species, especially *C. tropicalis* and *C. glabrata*. Notably, *C. glabrata* can acquire resistance to azoles and echinocandins as single-drug classes, as well as multidrug resistance involving all major drug classes. Azole resistance among *Candida* spp. involves several well-defined mechanisms, including upregulation of drug transporters, overexpression or alteration of the drug target, and cellular changes caused, in some cases, by nontarget effects induced by stress responses (Cowen et al., 2015; Perlin et al., 2017). Genetic factors, such as DNA repair, and chromosomal abnormalities help to induce drug-resistant phenotypes. Drug exposure also drives the emergence of resistance (Perlin et al., 2017). Numerous point mutations in *ERG11* have been reported in response to fluconazole. Investigation has shown that the *ERG11* mutation A395T was the major resistance mechanism, which was responsible for >83% of azole resistance in China (Fan et al., 2019). Echinocandin resistance is uncommon but is conferred by hot spot amino acid substitutions in glucan synthase. *Candida* isolates that are potentially echinocandin resistant or less susceptible often harbor amino acid substitutions in the Fks1p (and/or Fks2p in *C. glabrata*) gene (Alexander et al., 2013; Pham et al., 2014). Therefore, it is essential to screen the isolates with drug MIC values in the intermediate or resistant range to determine the mutations in *FKS1* and *FKS2* genes based on our surveillance data and compare the changes in MIC values caused by those mutations. Susceptibility testing of azoles and echinocandins, detection of the molecular mechanism of resistance, and combined antifungal treatment outcomes will allow more informed determinations of the value of these drugs in the antifungal armamentarium.

Our study has some limitations. First, this was a single-center retrospective study, so our results may not be generalizable to all patients with candidiasis and extrapolated to other institutions as the epidemiology of candidiasis can be highly institution specific. Second, this was a laboratory-based study, more detailed demographic and clinical characteristics were not collected, but it is the intention to include the collection of such data prospectively from this time forward. Nevertheless, this study provides important epidemiological findings which are instrumental in designing strategies for better management of candidiasis in our hospital.

In conclusion, our study has provided important updated information at a tertiary hospital in China on species distribution and antifungal susceptibility of yeast species. Over the 10-year surveillance, non-*C. albicans* species have become the predominant cause of nosocomial candidemia, and azole resistance is notable among *C tropicalis* and *C. glabrata* isolates. Multidrug resistance has emerged in *C. glabrata*, while overall resistance to echinocandins and flucytosine remains rare. Amphotericin B demonstrated excellent activity against all *Candida* species over the years. Continued surveillance, especially of national antifungal susceptibility trends, is warranted. The epidemiological findings in our study will pave the way for more in-depth studies and help us establish better antifungal stewardship in our hospital.

## Data Availability Statement

All datasets generated for this study are included in the article/Supplementary Material.

## Ethics Statement

All the strains isolated from patients were preserved in the Research Center for Medical Mycology at Peking University First Hospital. We conducted a retrospective study on the isolates and patient data collection including age and gender from electronic laboratory records. The study was approved by the ethics committee of Peking University First Hospital. The need for informed consent was waived by the Clinical Research Ethics Committee.

## Author Contributions

RL designed the experiments and supervised the data analysis. YS performed the antifungal susceptibility testing and wrote the manuscript. YS, XC, ZW, YY, and WL collected and analyzed the data. All authors discussed the results and commented on the manuscript.

## Conflict of Interest

The authors declare that the research was conducted in the absence of any commercial or financial relationships that could be construed as a potential conflict of interest.
