# Supplementary material for: Prevalence and Antifungal Susceptibility of Pathogenic Yeasts in China: A 10-Year Retrospective Study in a Teaching Hospital
Source: Front Microbiol. 2020 Jul 3;11:1401. doi: 10.3389/fmicb.2020.01401 (PMC7347963; doi:10.3389/fmicb.2020.01401)
Supplement: Supplementary file 1 [file Data_Sheet_1.pdf]

Table S1 Epidemiological cutoff values and clinical breakpoints for systemically active antifungal agents and *Candida* spp. determined by 24-h CLSI broth microdilution and SYO methods

**Organism**	**Antifungal agent**	**ECV (g/ml)**		**CBP (g/ml)**				**Reference**
		**WT**	**Non-WT**	**S**	**SDD**	**I**	**R**	
C. albicans	Amphotericin B	≤2	>2	—	—	—	—	[3]
	Flucytosine	≤1	>1	—	—	—	—	[4]
	Fluconazole	—	—	≤2	4	—	≥8	[1]
	Itraconazole	≤0.12	>0.12	—	—	—	—	[2]
	Posaconazole	≤0.06	>0.06	—	—	—	—	[3]
	Voriconazole	—	—	≤0.12	—	0.25–0.5	≥1	[1]
	Anidulafungin	—	—	≤0.25	—	0.5	≥1	[1]
	Caspofungin	—	—	≤0.25	—	0.5	≥1	[1]
	Micafungin	—	—	≤0.25	—	0.5	≥1	[1]
C. glabrata	Amphotericin B	≤2	>2	—	—	—	—	[3]
	Flucytosine	≤0.25	>0.25	—	—	—	—	[4]
	Fluconazole	—	—	—	≤32	—	≥64	[3]
	Itraconazole	≤4	>4	—	—	—	—	[3]
	Posaconazole	≤1	>1	—	—	—	—	[3]
	Voriconazole	≤0.25	>0.25	—	—	—	—	[3]
	Anidulafungin	—	—	≤0.12	—	0.25	≥0.5	[1]
	Caspofungin	—	—	≤0.12	—	0.25	≥0.5	[1]
	Micafungin	—	—	≤0.06	—	0.12	≥0.25	[1]
C. parapsilosis	Amphotericin B	≤2	>2	—	—	—	—	[3]
	Flucytosine	≤0.5	>0.5	—	—	—	—	[4]
	Fluconazole	—	—	≤2	4	—	≥8	[1]
	Itraconazole	≤0.5	>0.5	—	—	—	—	[2]

	Posaconazole	≤0.25	>0.25	—	—	—	—	[3]
	Voriconazole	—	—	≤0.12	—	0.25–0.5	≥1	[1]
	Anidulafungin	—	—	≤2	—	4	≥8	[1]
	Caspofungin	—	—	≤2	—	4	≥8	[1]
	Micafungin	—	—	≤2	—	4	≥8	[1]
C. tropicalis	Amphotericin B	≤2	>2	—	—	—	—	[3]
	Flucytosine	≤0.5	>0.5	—	—	—	—	[4]
	Fluconazole	—	—	≤2	4	—	≥8	[1]
	Itraconazole	≤0.5	>0.5	—	—	—	—	[3]
	Posaconazole	≤0.12	>0.12	—	—	—	—	[3]
	Voriconazole	—	—	≤0.12	—	0.25–0.5	≥1	[1]
	Anidulafungin	—	—	≤0.25	—	0.5	≥1	[1]
	Caspofungin	—	—	≤0.25	—	0.5	≥1	[1]
	Micafungin	—	—	≤0.25	—	0.5	≥1	[1]
C. krusei	Amphotericin B	≤2	>2	—	—	—	—	[3]
	Flucytosine	≤32	>32	—	—	—	—	[4]
	Fluconazole	IR	IR	IR	IR	IR	IR	[1]
	Itraconazole	≤1	>1	—	—	—	—	[3]
	Posaconazole	≤0.5	>0.5	—	—	—	—	[3]
	Voriconazole	—	—	≤0.5	—	1	≥2	[1]
	Anidulafungin	—	—	≤0.25	—	0.5	≥1	[1]
	Caspofungin	—	—	≤0.25	—	0.5	≥1	[1]
	Micafungin	—	—	≤0.25	—	0.5	≥1	[1]
C. lusitaniae	Amphotericin B	≤2	>2	—	—	—	—	[4]
	Flucytosine	≤0.5	>0.5	—	—	—	—	[4]
	Fluconazole	≤1	>1	—	—	—	—	[3]

	Itraconazole	≤1	>1	—	—	—	—	[3]
	Posaconazole	≤0.06	>0.06	—	—	—	—	[3]
	Voriconazole	≤0.03	>0.03	—	—	—	—	[2]
	Anidulafungin	≤1	>1	—	—	—	—	[3]
	Caspofungin	≤1	>1	—	—	—	—	[5]
	Micafungin	≤0.5	>0.5	—	—	—	—	[3]
C. guilliermondii	Amphotericin B	≤2	>2	—	—	—	—	[4]
	Flucytosine	≤1	>1	—	—	—	—	[4]
	Fluconazole	≤8	>8	—	—	—	—	[3]
	Itraconazole	≤1	>1	—	—	—	—	[2]
	Posaconazole	≤0.5	>0.5	—	—	—	—	[3]
	Voriconazole	≤0.5	>0.5	—	—	—	—	[2]
	Anidulafungin	—	—	≤2	—	4	≥8	[1]
	Caspofungin	—	—	≤2	—	4	≥8	[1]
	Micafungin	—	—	≤2	—	4	≥8	[1]
C. kefyr	Fluconazole	≤1	>1	—	—	—	—	[5]
	Posaconazole	≤0.25	>0.25	—	—	—	—	[5]
	Voriconazole	≤0.015	>0.015	—	—	—	—	[5]
	Anidulafungin	≤0.25	>0.25	—	—	—	—	[5]
	Caspofungin	≤0.03	>0.03	—	—	—	—	[5]
	Micafungin	≤0.12	>0.12	—	—	—	—	[5]

Note:

ECVs, epidemiological cutoff values; CBPs, clinical breakpoints; WT, wild type; non-WT, non-wild type; S, susceptible; SDD, susceptible, dose dependent; I, intermediate; R, resistant.

"—": not available; IR: intrinsic resistance.

References

1. CLSI. M60. Performance standards for antifungal susceptibility testing of yeasts, 1st edition. 2017 Clinical and Laboratory Standards Institute, Wayne, PA).

2. Espinel-Ingroff A, Turnidge J, Alastruey-Izquierdo A, et al. Method-Dependent Epidemiological Cutoff Values for Detection of Triazole Resistance in *Candida* and *Aspergillus* Species for the Sensititre YeastOne Colorimetric Broth and Etest Agar Diffusion Methods. Antimicrob Agents Chemother 2019; 63.

3. CLSI. M59. Epidemiological cutoff values for antifungal susceptibility testing, 2nd Edition., 2018 Clinical and Laboratory Standards Institute, Wayne, PA).

4. Canton E, Peman J, Hervas D, et al. Comparison of three statistical methods for establishing tentative wild-type population and epidemiological cutoff values for echinocandins, amphotericin B, flucytosine, and six *Candida* species as determined by the colorimetric Sensititre YeastOne method. J Clin Microbiol 2012; 50:3921-6.

5. Pfaller, M. A., and Diekema, D.J. (2012). Progress in antifungal susceptibility testing of *Candida* spp. by use of Clinical and Laboratory Standards Institute broth microdilution methods, 2010 to 2012. *J. Clin. Microbiol.* 50, 2846-2856. doi: 10.1128/JCM.00937-12

Table S2. Species distribution of yeast isolates over 10 years of surveillance

Species	Overall	2010	2011	2012	2013	2014	2015	2016	2017	2018	2019
	No. (%)	No (%)	No. (%)	No. (%)	No. (%)	No. (%)	No. (%)	No. (%)	No. (%)	No. (%)	No. (%)
C. albicans	259 (49.2)	20 (35.1)	39 (50.0)	33 (49.3)	12 (30.8)	18 (35.3)	30 (58.8)	11 (47.8)	15 (44.2)	34 (53.1)	47 (73.4)
C. parapsilosis sensu stricto	91 (17.3)	14 (24.6)	10 (12.8）	17 (25.4)	14 (35.9)	8 (15.7)	4 (7.8)	4 (17.4)	10 (29.4)	8 (12.5)	2 (3.1)
C.tropicalis	73 (13.9)	12 (21.1)	11 (14.1)	6 (9.0)	8 (20.5)	5 (9.8)	5 (9.8)	2 (8.7)	4 (11.8)	14 (21.9)	6 (9.3)
C. glabrata	52 (9.9)	9 (15.8)	14 (17.9)	3 (4.5)	5 (12.8)	11 (21.6)	4 (7.8)	3 (13.0)	1 (2.9)		2 (3.1)
Cryptococcus neoformans	14 (2.7)	2 (3.5)	1 (1.3)	3 (4.5)		2 (3.9)	2 (3.9)		1 (2.9)	2 (3.1)	1 (1.6)
C. krusei	12 (2.3)		1 (1.3)	2 (3.0)			1 (2.0)	2 (8.7)	1 (2.9)	4 (6.3)	1 (1.6)
Trichosporon asahii	4 (0.8)					2 (3.9)	1 (2.0)			1 (1.6)	
Rhodotorula glutinis	4 (0.8)		1 (1.3)			1 (2.0)					2 (3.1)
C. lusitaniae	3 (0.6)			2 (3.0)				1 (4.4)			
C. guilliermondii	3 (0.6)		1 (1.3)				1 (2.0)				1 (1.6)
C. inconspicua	3 (0.6)					1 (2.0)				1 (1.6)	1 (1.6)
Pichia anomalus	2 (0.4)						2 (3.9)				
C. metapsilosis	1 (0.2)								1 (2.9)		
C. utilis	1 (0.2)								1 (2.9)		
C. lipolytica	1 (0.2)			1 (1.5)							
C. kefyr	1 (0.2)						1 (2.0)				
Cryptococcus curvatus	1 (0.2)					1 (2.0)					
C. carpophila	1 (0.2)										1 (1.6)
Trichosporon inkin	1 (0.2)					1 (2.0)					

Table S3. Yeast species recovered from clinical samples

N (%)	Blood	BALF	Ascitic fluid	Pleural fluid	Pus	CSF	Peritoneal dialysis	CVC	Bile	Hydrarthrosis	Tissue	Bone marrow
C. albicans	61 (33.2)	104 (72.7)	39 (48.8)	19 (48.7)	9 (40.9)	11 (57.9)	1 (6.3)	5 (55.6)	5 (62.5)	2 (66.7)	2 (66.7)	0
C. parapsilosis sensu stricto	48 (26.1)	8 (5.6)	9 (11.3)	5 (12.8)	7 (31.8)	1 (5.3)	11 (68.8)	1 (11.1)	0	1 (33.3)	0	0
C. tropicalis	26 (14.1)	17 (11.9)	15 (18.8)	6 (15.4)	2 (9.1)	0	2 (12.5)	2 (22.2)	1 (12.5)	0	1 (33.3)	0
C. glabrata	31 (71.2)	3 (2.1)	9 (11.3)	3 (7.7)	3 (13.6)	1 (5.3)	0	1 (11.1)	1 (12.5)	0	0	0
C. krusei	4 (2.2)	4 (2.8)	3 (3.8)	2 (5.1)	0	0	0	0	0	0	0	0
C. guilliermondii	1 (0.5)	1 (0.7)	1 (1.3)	0	0	0	1 (6.3)	0	0	0	0	0
C. inconspicua	0	0	0	2	0	0	0	0	11 (12.5)	0	0	0
C. lusitaniae	0	3 (2.1)	0	0	0	0	0	0	0	0	0	0
C. utilis	0	0	1 (1.3)	0	0	0	0	0	0	0	0	0
C. metapsilosis	0	0	1 (1.3)	0	0	0	0	0	0	0	0	0
R. glutinis	3 (1.6)	0	0	0	0	0	1 (6.3)	0	0	0	0	0
***Cryptococcus* spp.**	3 (1.6)	3 (2.1)	0	2 (5.1)	0	6 (31.6)	0	0	0	0	0	1
***Trichosporon* spp.**	2 (1.1)	0	1 (1.3)	0	1 (4.5)	0	0	0	0	0	0	0
**Other *spp.***	5 (2.7)	0	1 (1.3)	0	0	0	0	0	0	0	0	0
**In total**	184 (34.9)	143 (27.1)	80 (15.2)	39 (7.4)	22 (4.2)	19 (3.6)	16 (3.0)	9 (1.7)	8 (1.5)	3 (0.5)	3 (0.5)	1 (0.2)

Table S4. Yeast species isolated from candidemia patients

Species	2010	2011	2012	2013	2014	2015	2016	2017	2018	2019	Total (n, %)
C. albicans	2	13	6	5	5	7	2	4	12	5	61 (33.2)
C. parapsilosis sensu stricto	6	3	9	13	4	3	3	3	3	1	48 (26.1)
C. glabrata	6	9	1	2	9	2	1	0	0	1	31 (16.8)
C. tropicalis	4	3	2	5	3	2	1	1	4	1	26 (14.1)
C. krusei	0	0	1	0	0	0	1	0	2	0	4 (2.2)
Cryptococcus neoformans	0	0	0	0	2	1	0	0	0	0	3 (1.6)
Trichosporon asahii	0	0	0	0	2	0	0	0	0	0	2 (1.1)
Pichia anomalus	0	0	0	0	0	2	0	0	0	0	2 (1.1)
Rhodotorula glutinis	0	0	0	0	1	0	0	0	0	2	3 (1.6)
C. kefyr	0	0	0	0	0	1	0	0	0	0	1 (0.5)
C. lipolytica	0	0	1	0	0	0	0	0	0	0	1 (0.5)
C. carpophila	0	0	0	0	0	0	0	0	0	1	1 (0.5)
C. guilliermondi	0	0	0	0	0	0	0	0	0	1	1 (0.5)
Total	18	28	20	25	26	18	8	8	21	12	184

Table S5. Cross-resistance to triazoles in the 527 tested isolates

***Candida* species (number of isolates tested)**	**Number (%) of isolates with resistance or non-wild-type to indicated triazoles**
***C. albicans* (259)**	**12 (4.6)**
Fluconazole + itraconazole + posaconazole + voriconazole	4 (1.5)
Fluconazole + itraconazole + posaconazole	2 (0.8)
Itraconazole + posaconazole	6 (2.3)

***C. glabrata* (52)**	**23 (44.2)**
Fluconazole + itraconazole + posaconazole + voriconazole	4 (7.7)
Fluconazole + voriconazole + posaconazole	5 (9.6)
Fluconazole + voriconazole	5 (9.6)
Voriconazole + posaconazole	9 (17.3)
***C. tropicalis* (73)**	**24 (32.9)**
Fluconazole + posaconazole + voriconazole + itraconazole	15 (20.6)
Fluconazole + posaconazole + itraconazole	1 (1.3)
Fluconazole + posaconazole + voriconazole	4 (5.5)
Fluconazole + posaconazole	4 (5.5)
***C. krusei* (12)**	**1 (8.3)**
Fluconazole + posaconazole + voriconazole	1 (8.3)

Table S6. Resistance to antifungals of yeast isolates from fungemia patient

***Candida* species (n)**	**with resistance or non-wild-type to indicated antifungal n (%)**
***C. albicans* (61)**	**8 (13.1)**
Posaconazole	3 (4.9)
Anidulafungin + Micafungin	1 (1.6)
Fluconazole + posaconazole	1 (1.6)
Itraconazole + posaconazole	1 (1.6)

Caspofungin + Anidulafungin + Micafungin + Flucytosine + posaconazole	1 (1.6)
Fluconazole + itraconazole + posaconazole + voriconazole	1 (1.6)
C. glabrata **(31)**	**23 (74.2)**
Voriconazole	4 (12.9)
posaconazole + voriconazole	8 (25.8)
Fluconazole + voriconazole	3 (9.7)
Fluconazole + voriconazole + posaconazole	4 (12.9)
Fluconazole + voriconazole + posaconazole + itraconazole	3 (9.7)
Caspofungin + Anidulafungin + Micafungin	1 (3.2)
C. tropicalis **(26)**	**19 (73.1)**
Fluconazole	1 (5.3)
Posaconazole	8 (30.8)
posaconazole + voriconazole+itraconazole	2 (7.7)
Fluconazole + voriconazole + posaconazole + itraconazole	7 (25.5)
Fluconazole + voriconazole posaconazole + itraconazole + Caspofungin + Anidulafungin + Micafungin	1 (3.8)
C. parapsilosis sensu stricto **48)**	**2 (4.2)**
Fluconazole	1 (2.1)
Flucytosine	1 (2.1)
